# Inhibiting the Laydown of Polymeric Carbon and Simultaneously Promoting Its Facile Burn-Off during the Industrial-Scale Production of Hydrogen with Nickel-Based Catalysts: Insights from Ab Initio Calculations

**DOI:** 10.3390/nano13010040

**Published:** 2022-12-22

**Authors:** Aniekan Magnus Ukpong

**Affiliations:** 1 Theoretical and Computational Condensed Matter and Materials Physics Group (TCCMMP), School of Chemistry and Physics, University of KwaZulu-Natal, Pietermaritzburg 3201, South Africa; ukponga@ukzn.ac.za; Tel.: +27-033-260-5875; Fax: +27-031-260-3091; 2 National Institute for Theoretical and Computational Sciences (NITheCS), KwaZulu-Natal Node, Pietermaritzburg 3201, South Africa

**Keywords:** carbon laydown, burn-off, thermodynamic profile, density functional theory

## Abstract

This paper presents a computational study of the mechanistic models for the laydown of carbon species on nickel surface facets and the burn-off models for their gasification mechanism in methane steam reforming based on density functional theory. Insights into catalyst design strategies for achieving the simultaneous inhibition of the laydown of polymeric carbon and the promotion of its burn-off are obtained by investigating the influence of single atom dopants on nickel surfaces. The effects of single atom dopants on adsorption energies are determined at both low and high carbon coverages on nickel and used to introduce appropriate thermodynamic descriptors of the associated surface reactions. It is found that the critical size of the nucleating polymeric carbon adatom contains three atoms, i.e., C_3_. The results show that the burn-off reaction of a polymeric carbon species is thermodynamically limited and hard to promote when the deposited carbon cluster grows beyond a critical size, C_4_. The introduction of single atom dopants into nickel surfaces is found to modify the structural stability and adsorption energies of carbon adatom species, as well as the free energy profiles of surface reactions for the burn-off reactions when CH_4_, H_2_O, H_2_, and CO species react to form hydrogen. The results reveal that materials development strategies that modify the sub-surface of the catalyst with potassium, strontium, or barium will inhibit carbon nucleation and promote burn-off, while surface doping with niobium, tungsten, or molybdenum will promote the laydown of polymeric carbon. This study provides underpinning insights into the reaction mechanisms for the coking of a nickel catalyst and the gasification routes that are possible for the recovery of a nickel catalyst during the steam reforming of methane for large-scale production of hydrogen.

## 1. Introduction

Producer gas (also known as synthesis gas or syngas) plays a central role in the large-scale production of the hydrogen required to generate clean energy. Syngas is a variable mixture of hydrogen and carbon monoxide. It can be produced from a variety of different carbon-based materials. These include biomass (wood gas), plastics, coal, municipal waste, etc. The industrial production of hydrogen utilizes the methane steam reforming (MSR) reaction [1,2] or methane dry reforming by carbon dioxide reduction [3,4]. These two reactions are typically implemented as routes in the large-scale production of syngas. These routes involve independent reactions for the steam reforming of methane [CH_4_(g) + H_2_O(g) → CO(g) + 3H_2_(g)] and the dry reforming of methane [CO_2_(g) + CH_4_(g) → 2CO(g) + 2H_2_(g)] over supported Ni-catalyst. Even though the dry-reforming reaction is energetically far less intensive compared to the steam-reforming reaction, the Ni-based catalyst used in both reactions are susceptible to carbon build-up, which eventually cokes and deactivates the catalyst [5,6]. The steam reforming of methane is a highly endothermic but reversible reaction. As such, heat must be supplied to the process for the reaction to proceed. However, a water gas shift reaction can be initiated by allowing the on-stream reaction between the carbon monoxide with steam over a suitable precious metals (PGMs)-based catalyst [7,8] to produce carbon dioxide and more hydrogen [CO(g) + H_2_O(g) → CO_2_(g) + H_2_(g)]. For the dry-reforming reaction, the supported Ni-catalyst is believed to deactivate easily when compared to the PGMs-based alternatives (e.g., Pt, Pd) due exclusively to the excessive laydown of carbon [9].

A major thrust of contemporary industrial research is to develop an underpinning understanding of the carbon laydown and its associated burn-off mechanisms. This is aimed primarily at gaining insights into the possible mechanisms that could be integrated on-stream to make it unfavorable for the polymeric carbon formation on catalyst surface. It is shown here that surface modification by dopants can inhibit the carbon laydown either by promoting a facile burn-off or by disfavoring the nucleation and concatenation of carbon. Consider that the reaction of methane with high-pressure steam over the surface of alumina-supported Ni catalysts during the MSR reaction involves the simultaneous laydown and burn-off of the carbon. However, if the thermodynamics of the reaction does not favor a fast conversion of the surface carbon adatom to carbon monoxide for burn-off, then sufficient carbon species can nucleate, grow, and polymerize to ultimately form coke. Thus, the optimization of the catalysts’ surface or the process conditions is necessary for carbon burn-off, which is necessary to avoid coking. It is also necessary to explore auxiliary reactions that can promote the gasification of any excess carbon to remove them from the catalyst surface. This is important because the high operating temperature of the reaction favors the formation of carbon deposits. Since MSR is sensitive to factors, such as the operating temperature of the reformer tube, pressure drop, and the carbon formation, a good understanding of carbon-mediated surface reactions could allow for process optimization and improved management of the energy outlay of the process. 

Despite the importance of coking in heterogenous catalysis, not much have been reported in the literature on the reaction thermodynamics and kinetics of the laydown and burn-off mechanisms of polymeric carbon on nickel surface. Experimental data on the related surface reactions are also scarce although their underpinning understanding is required for the development of chemical process technologies. To our knowledge, empirical scientific data are only available for carbon–metal binding energies on Ni(111) and Ni(100) single crystal surfaces. Coke is the carbon deposit that forms on the surface facets of a supported nickel catalyst due to the hydrocarbon decomposition reactions that occur on the surface of the catalyst [10]. The nucleation of carbon on the surface of a real catalyst is known to cause local hot spots, breakage of catalyst particles, and blockage of the reactor tube. These poison the active sites, which leads ultimately to the deactivation of the catalyst [11]. Carbon can deposit easily on metal catalyst surfaces if their acidity is high [12]. Unwanted carbon deposits on the catalyst can also manifest in MSR as coke. Carbon deposits also have the catastrophic effect of metal dusting corrosion of the steam reformer tube [13,14]. Since the active sites on a catalyst surface can be regulated by the size and morphology of the active species of the metal nanoparticle to enhance the catalytic performance and effectiveness, it is possible to tailor the chemical activity to make it unfavorable towards reactions that lead to the formation of coke deposits on the catalyst surface.

Herein, an investigation of the reaction mechanisms involved in methane steam reforming is presented to understand its unwanted side reactions and to gain insights into how to prevent them from forming unwanted carbon deposits. First-principles electronic structure calculations are performed to show that suitable doping nickel surfaces can modify the intrinsic affinity towards carbon adsorption, making it unfavorable towards the formation of carbon deposits. Insights from this study are used to propose atomic-level modifiers that can serve either as inhibitors of carbon formation or promoters of carbon gasification on the nickel catalyst surface. The effects of single-atom modification of different surface facets of a typical Ni catalyst on the sequential laydown and burn-off of monomeric and polymeric carbon is elucidated. It is found that when the nucleating polymeric carbon reaches a critical size of C_4_, gasification becomes thermodynamically limited and hard to promote because the deposited carbon fragment is energetically favored to concatenate. Correlations are established between surface adsorption descriptors and structural stability metrics of yielding insights into the thermodynamics of carbon gasification at sufficiently high temperature. The results reveal that materials development strategies that modify the catalyst surface with potassium, strontium, or barium will lead to the inhibition of carbon nucleation, while modification with niobium and molybdenum will favor carbon laydown. The interrelationship between adsorption energies of monomeric and polymeric carbon species during the carbon laydown is used to establish the effect of carbon cluster size, benzenic ring formation, and carbon branching on the thermodynamics of catalyst coking and its gasification potential. 

This paper is organized as follows: The computational details and structural models used to develop a rational understanding of the simultaneous inhibition of the laydown of polymeric carbon and promotion of the facile burn-off reaction in methane steam reforming is provided in Section 2. Top- and sub-surface modification by dopant species are also considered. In addition, details of the first principles calculations are also provided in Section 2. In Section 3, a mechanistic model of carbon laydown on nickel catalysts is presented in Section 3.1. Correlations are also established between the free energy of nickel surfaces and the carbon adsorption energy in Section 3.2. The thermodynamic framework for the rational promotion of carbon burn-off in methane steam reforming is presented in Section 3.3. Finally, conclusions are drawn in Section 4.

## 2. Computational Details 

The nickel surfaces were modelled in the slab approximation of the periodic supercell model. The Ni(111), Ni(211), and Ni(322) steps each contained 5 atomic layers. The free energy *γ* of a given Ni surface slab with two equivalent surfaces on both sides and a thickness *L* was calculated using the expression [15]:

(1) $$\gamma =\frac{1}{AL}\left(E^{slab}-NE^{bulk}\right)$$

where $E^{slab}$ denotes the total energy of the slab in a supercell, *N* is the total number of atoms in the slab, *A* is the area of the bottom or top face of the slab, and $E^{bulk}$ denotes the energy per atom in the bulk crystal. The slab thickness *L* is in units of atomic layers or monolayers (MLs).

The 4 × 4 × 1 supercell was used to model the surface layers. This supercell size is large enough for the adsorbed systems considered. These cell parameters were kept fixed in the local minimum structures throughout the calculations. The image optimization was carried out using the atomic simulation environment (ASE) version 3.22.1 (Computational Atomic-scale Materials Design, Denmark) [16]. Only the atomic positions within the two topmost atomic layers were relaxed, while atomic positions in the last two atomic layers were kept fixed at their positions in fcc Ni. In all the calculations, the middle atomic layer of the Ni surface is allowed to relax fully. Position relaxation calculations for the geometry optimization were performed using the conjugate gradient scheme until the total energies were converged to 10^−6^ eV, and the forces on each atom converged to less than 10^−3^ eV/Å. The effect of adding a single atom dopant to the surface of the nickel catalyst was investigated by studying the electronic effects of inserting a dopant at the top- and sub-surface positions in the step surface facet. This allows for simulations of the disruptive effects of dopants on the adsorption energy of carbon species. Geometric optimization and vibrational frequency analysis of the surface adsorbates was carried out to determine the Gibbs free energy of the adsorption model. 

Spin polarized first-principles calculations were performed within the framework of density functional theory (DFT) using the svdW-DF2 functional, as implemented in the grid-based projector-augmented wave (GPAW) code version 22.8.0 (Computational Atomic-scale Materials Design, Denmark) [17,18]. The svdW-DF2 functional denotes the spin-polarized form of the second version of the Dion et al. model [19] of the van der Waals density functional (vdW-DF2) proposed by Lee et al. [20]. The exchange-correlation potential is treated in the generalized gradient approximation (GGA), since the vdW-DF2 functional uses the refined version of the Perdew and Wang (PW86) functional as the gradient correction on exchange [21,22], in combination with a nonlocal correction for dispersive interactions, for which Z_ab_ = −1.887 [23]. Interactions between valence electrons and ion cores are described using projector-augmented wave (PAW) potentials [24,25], and the kinetic energy was expanded in the plane waves basis with a cut-off of 600 eV. The electronic energy was converged to within 10^−6^ Ry. In the self-consistent calculations of energies, electron states were populated using Fermi–Dirac scheme [26]. The calculations were fully converged at the smearing width of 0.005 eV.

The adsorption energy (E_ad_) of a carbon fragment on a nickel surface was obtained by calculating the total energy for the isolated slab and for the isolated molecule. The adsorbate was then added to the slab and relaxed, and the total energy for this composite system was calculated. The adsorption energy was obtained as the sum of the isolated energies minus the energy of the composite system. The adsorption energy was computed at different high symmetry adsorption sites using the following relationship:

E_ad_ = E_AB_ − (E_A_ + E_B_), (2)

where E_AB_ denotes the total energy of the adsorption complex, E_A_ represents the energy of an isolated carbon cluster (C_n_), and E_B_ denotes the total energy of the pristine surface. In each case, both energies are calculated using the same supercell and DFT-tuning parameters. To provide the basis for a rational understanding of how the carbon adsorption may be used to develop a mechanistic model for the catalyst coking and polymeric carbon burn-off, the thermodynamic stability of the different step and terrace surfaces of nickel were established first at 0 K and then thermodynamics corrections for the effect of finite temperature were included at 973.15 K. 

The effect of temperature on the reactants and products of the reaction models were included by incorporating thermodynamic corrections at finite temperature (*T*) and pressure (*P*) in which the free energies of reaction were calculated using statistical thermodynamics. The Gibbs free energy of the gas-phase species (*x*) is given by $G_{T,P}^{x}=E_{DFT}^{x}+E_{ZPE}-\Delta H^{0,T}-{TS}^{T}+k_{B}\ln\;\left(P/P^{0}\right)\;$where $E_{DFT}^{x}$ denotes the 0 K total energy from *DFT*,${\;E}_{ZPE}$ is the vibrational zero-point energy, $\Delta H^{0,T}$ is the change in enthalpy when the temperature is raised from 0 K to a finite temperature (*T*) and $S^{T}$ is the entropy at *T*, in each case, where the pressure of gas phase species *P* is set to *P*^0^ (i.e., *P* = 1.0 bar) where *k_B_* is Boltzmann’s constant. The internal electronic energy E of the adsorbed carbon fragment (*z*) on a nickel surface facet (*i*) was obtained using $\Delta E_{DFT}^{z}=E_{i}^{z}-E_{i}$ where$\;E_{i}^{z}\;$and $E_{i}$ denote the *DFT* energy of the nickel surface with and without the adsorbate, respectively. The change in enthalpy was replaced by the change in internal energy $\Delta U^{0,T}$. Thus, the free energy of the adsorbed species is $G_{T,P}^{z}=E_{DFT}^{z}+E_{ZPE}+\Delta U^{0,T}-TS^{T}$.

Contributions to the free energy from low-frequency modes of vibration and from internal molecular rotation around bonds were computed at 700 °C within the harmonic approximation, and gas phase species were treated as ideal gases. This approach to the computational modelling of the thermodynamic reaction profile can capture the essentials of the methane steam reforming reaction on nickel catalyst surfaces—especially after corrections for van der Waals interactions have been included due to the adsorbate surface chemisorption. This computational model is consistent with the one used recently to study the reaction profiles of propane dehydrogenation to propene on defective graphene [27].

## 3. Results and Discussion

### 3.1. Mechanistic Model of Carbon laydown on Nickel Catalysts

#### 3.1.1. Nucleation of Carbon Clusters from Methane Decomposition

Hereunder, a mechanistic model is first developed for understanding the single-atom nucleation of carbon species and the growth of polymeric carbon on the surface of nickel catalysts. The adsorption energy of the carbon adatom was evaluated at high symmetry adsorption sites of the Ni(211) step surface at the step hollow (SH), step bridge (SB), terrace atop (TA), and terrace hollow (TH) sites. The sequential laydown of carbon adatoms on the SH site is described using the larger Ni(322) step surface as the preferred adsorption model. This is crucial because the adsorption of single carbon atoms on the SH site is the most favorable adsorption energy, whereas the size of the Ni(211) step surface is too small to describe the larger fragments of polymerized carbon, which arise due to carbon concatenation and the formation of branched carbon chains.

The reaction of the methane gas with the nickel catalyst is assumed to occur at the most preferred high-symmetry adsorption (i.e., SH) site of the nickel surface for the nucleation of carbon species is understandable as a sequential process wherein a single carbon adatom is adsorbed at energetically preferred sites. Here, it is assumed that the reaction for the formation of surface carbon is via irreversible decomposition of methane via:

CH_4_(g) + * → C*(s) + 2H_2_(g), (3)

where the asterisk (*) denotes the SH site and C* denotes the adsorbed carbon. The results presented herein are from calculations performed under the assumption that there is no recombination of the surface species, C* + 4H*, after hydrogen stripping from methane. The adsorption strengths at the four high symmetry sites available for the carbon monomer (C_1_) adsorption on the Ni(211) step surface rank as follows: SH > SB > TA > TH. Thus, the nucleation of the monomer occurs favorably at the SH site with an adsorption energy of 0.95 eV. Thus, carbon laydown starts as a preferential nucleation of the C_1_ species at the SH site. 

**Figure 1:**
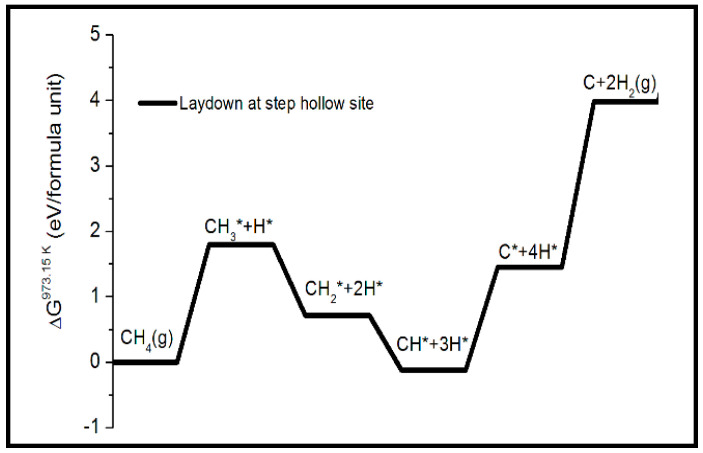
Thermodynamic reaction profile of the methane decomposition reaction on the Ni(322) surface for the laydown of monomeric carbon at the step hollow site of the surface.

Figure 1 shows the thermodynamic reaction profile for the laydown of a single carbon on Ni(322) surface at 973.15 K from the decomposition of methane. This shows that the first intermediate step that involves the stripping reaction for the removal of the surface hydrogen species from the methane to form the surface radical species (H*) is unfavorable, relative to the second and third intermediate reaction steps. By contrast, the fourth intermediate step of the hydrogen stripping reaction for the formation of four radical H* species on the surface is energetically less favorable than the previous (i.e., third step) by over 1.5 eV per formula unit (f.u.). The eventual reaction for the desorption of molecular H_2_ species and the laydown of the first carbon adatom on the SH site is over 2 eV/f.u. more unfavorable than the previous intermediate step. This endothermic reaction has an enthalpy change ΔH of 75 kJ/mol at 298 K [28].

Figure 1 shows that the methane cracking reaction for the laydown of carbon at 700 °C is also endothermic. Thus, the reaction does not proceed spontaneously and must be activated catalytically. This insight also offers the first clues to the realistic possibility of thermodynamically disfavoring the methane cracking reaction by manipulating the catalyst surface. It is shown in Section 3.2 that the addition of alkali metal atoms, such as K, Cs, and Rb, as top-surface (or sub-surface) dopants, has a strong influence on the surface energy. This effect is directly correlated with an increase (or decrease) in the adsorption energy of the single carbon adatom by making it either favorable or unfavorable for the nucleation to proceed.

#### 3.1.2. Understanding the Growth of Polymeric Carbon on Nickel Surfaces

Polymeric carbon is composed of several atoms of elemental carbon, arranged in sp² orbitals, forming graphitic layers of hexagonal planes with different degrees of long and short-ranged order [29]. To couple an isolated carbon adatom to an existing polymeric carbon fragment, it is assumed that chain growth does not consume the Ni surface steps after initial carbon nucleation. As the polymeric carbon laydown progresses sequentially towards monolayer coverage, the Ni(322) step model is no longer suitable for describing the carbon laydown as polymeric at full monolayer coverage with graphitic carbon. Hence, the Ni(111) model of the terrace surface is used instead to understand the growth of polymeric carbon as a model for catalyst coking. The polymerization of the carbon monomers is described herein as a reversible addition of a monomer species to an existing polymeric chain on the surface according to the sequential reactions:

C_1_* + * ↔ [C_1_]* + * (4)

C_1_* + C_1_* ↔ [C_2_]* + * (5)

C_2_* + C_1_* ↔ [C_3_]* + * (6)

… … …

C_n_* + C_1_* ↔ [C_n + 1_]* + * (7)

An exhaustive adsorption of single carbon adatoms on all available SH sites of the Ni(322) surface is energetically more favorable compared to the formation of the dimer or linear chains. We, therefore, investigate the effects of branching and ring formation on polymerization. These are important, since the uninhibited growth of a nucleating carbon results in the formation of linear and branched carbon chains and the formation of hexagonal rings.

**Figure 2:**
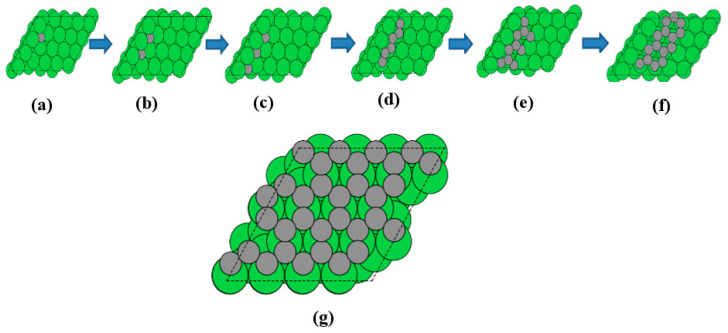
Sequential adsorption of carbon monomers for the nucleation of monomeric species on the step hollow site (**a**–**c**) and the growth of polymeric species as linear (**d**), branched (**e**), and hexagonal ring (**f**) species. Polymeric carbon laydown modelled as an adsorption graphene overlayer on the Ni(111) surface (**g**).

Figure 2a–f show the sequential adsorption of carbon monomers for formation and growth of polymeric species from the SH site on the Ni(322) step surface. This model is used to understand the role of carbon catenation, including the formation of linear chain, branching, and hexagonal rings in the sequential growth of polymetric carbon species. Thus, whenever there are non-adsorbed SH sites still available on the Ni step surface, the carbon will continue to nucleate from isolated monomeric species. In the following ball-and-stick models, it is shown that the interaction between a given C*_n_* cluster species and the Ni surface terrace influences the laydown of polymeric carbon by favoring the formation of branched species instead of linear chains. As a result, the laydown of carbon at full ML coverage is shown in Figure 2g as the model of a graphene overlayer on the flat Ni(111) surface. This is a suitable model for understanding the early stages of nickel catalyst coking in the MSR.

#### 3.1.3. Thermodynamics of Carbon Chains Branching during Polymeric Carbon Laydown

**Figure 3:**
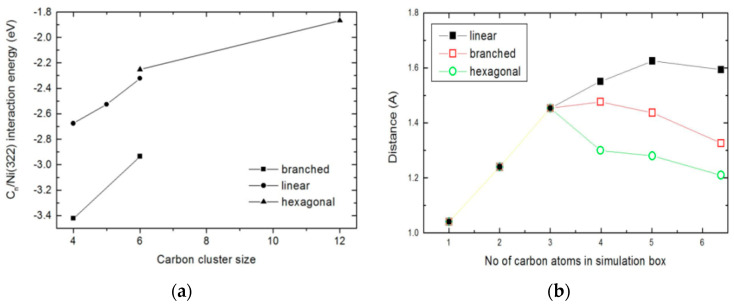
Dependence of the binding energy of a growing carbon cluster on local geometry (**a**) and the dependence of the variation in the separation distance between the nucleated carbon cluster and the Ni(322) step surface on number of carbons in the cluster (**b**).

Figure 3 shows the changes in binding energy between the Ni(322) surface and a growing carbon cluster with the local geometry of the cluster size (C*_n_*). Figure 3a shows the effect of carbon branching within the concatenated chain on the thermodynamics of the laydown process. Figure 3a shows that carbon fragments adsorbed in branched geometry exhibit a stronger binding energy with the step surface relative to the linear carbon fragments. Figure 3b shows the cluster binding distance between the adsorbed carbon cluster and the Ni(322) step surface. For carbon clusters that are nucleated with a size *n* ≤ 3, the binding distance between the cluster and the surface shows a linear dependency. However, Figure 3b shows that as the number of carbon increases in the deposited carbon beyond *n* = 3, and the interaction between the cluster and the step surface changes significantly depending on the cluster geometry. Overall, the cluster/surface separation distances (*h*) saturate at C_3_, and this corresponds to the onset of ring formation. Our analysis shows that the distance *h* increases with the number of carbon species *n* in the nucleating carbon cluster C*_n_* the up to C_5_ (C_4_) in linear (branched) chains.

The interaction between the carbon cluster C_n_ and nickel surface facets Ni(ijk) is crucial for understanding the thermodynamic process of carbon clustering. In the linear chain, for instance, the dependence of the binding distance with carbon cluster size shows a linear increase up to a chain size of *n* = 5 before it decreases. The interaction between the carbon deposit and the surface progressively weakens in linear carbon chains that contain more than five carbons. The strongest interaction (i.e., shortest interaction distance) is obtained when the hexagonal ring cluster starts to form. The thermodynamic profile of carbon branching suggests that a catalyst development strategy can be designed to stop coke formation by targeting the inhibition of the early-stage carbon laydown mechanism. This is advantageous over a catalyst design strategy that focuses on the later stages of the coking wherein branching has completed and hexagonal rings of the graphene-like structure has formed already. 

The variations in the carbon cluster/surface interaction distance (*h*) with the cluster size (estimated herein in terms of the number of carbon atoms *n* in cluster C*_n_*) contains important insights on the carbon–carbon concatenation process. There is a critical nucleation size of three atoms, beyond which the stability of the nucleating adatoms has an explicit sensitivity to the local geometry, as can be seen in Figure 3b. The interaction energy between the Ni(322) surface and the cluster increases linearly as the cluster size increases. As the polymeric carbon laydown progresses and the size of the C*_n_* species grows, the separation distance between the carbon fragment and the Ni(322) step surface saturates in the linear chain when *n* = 5. With the nucleation of branched and hexagonal ring clusters, the interaction distance saturates when the cluster size is *n* = 3 and *n* = 4, respectively. 

Beyond these saturation interactions, any increase in the size of a cluster leads to a significant reduction in the fragment binding distances. The reduced binding distances of carbon fragments suggest that delaying the inhibition of carbon laydown long enough for branched and hexagonal-ringed carbon clusters to form is not ideal for implementing an early-stage coking inhibition strategy. Taken together, the following insights are obtained. It is more favorable to first fill-up all the step hollow sites, before the next available step Ni atop sites are filled in linear geometry. Branching is energetically unfavorable unless all step sites are filled. It is preferable to form a seven-atom linear chain than a branched chain with an unclosed six-membered ring containing equal number of atoms.

### 3.2. Correlations between the Free Energy of Nickel Surfaces and The Carbon Adsorption Energy

#### 3.2.1. Effect of Dopants on the Thermodynamics of Coking

The scale of the coking problem of nickel catalysts has made it an imperative task of both scientific and industrial importance to develop a rational strategy of inhibition. Herein, a deep understanding of coking is gained within the framework of non-equilibrium thermodynamics of the reacting species using surface descriptors. This is crucial for inhibiting the coke deposits that form in nickel catalyst in MSR reactions. This is because at the high operating temperatures involved, the activity of the deposited carbon species in coke is high enough to cause the catastrophic corrosion of the steam reformer tube. So far, it is intuitive that the formation of the branched and hexagonal-ringed carbon clusters on the nickel surface will play nontrivial roles in the thermodynamics of the coking of the nickel catalyst. The interrelationships between the surface energy, adsorption energy, and the binding energy of surface dopants are analyzed and used as thermodynamic descriptors of the coking of nickel surfaces, which are presented in the following paragraph to gain insights into carbon laydown inhibition. 

**Figure 4:**
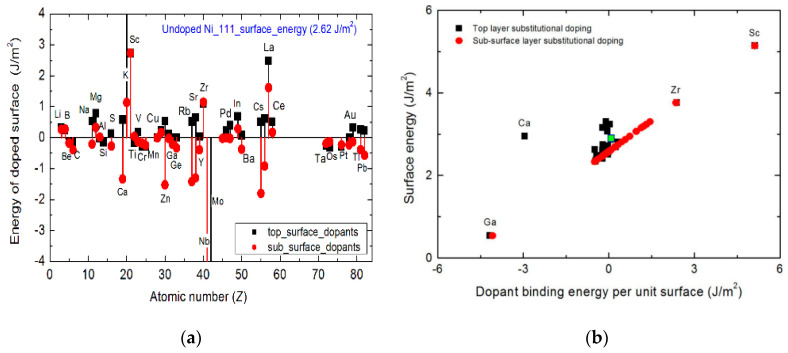
Effects of single-atom dopants on the free energy of the Ni(111) surface for top-surface (black squares) and sub-surface (red spheres) doping (**a**). Correlation between the surface energy and the dopant binding energy per unit area in the absence of the carbon adsorbate (**b**). The changes in the surface energy with doping are determined relative to the free energy undoped surface, as indicated by the green square.

Figure 4 shows the effect of introducing a single-atom dopant on the Ni(111) surface energy and the dopants effects on the correlation between the surface energy and the dopant-binding energy per unit area in the absence of the carbon adsorbate. A dopant is introduced into the top- or sub-surface. The disruptive changes in free energy are calculated relative to the free energy of the undoped surface. The dopant-induced variations in the surface energy suggest that the introduction of a dopant species influences the surface sensitivity by altering the surface energy—even before an adatom of carbon is adsorbed. The thermodynamic free energy of the pristine Ni(111) surface is 2.62 Jm^−2^. This surface energy is consistent with results of another related DFT calculation [30]. Crucially, Figure 4a shows that the position of the introduced dopant and its chemical identity have non-trivial influences on the surface free energy.

The addition of Mo (or Nb) as a dopant in the top-surface (or sub-surface) position is found to make the nickel surface become more thermodynamically stable, relative to the undoped surface. This characterization is because the surface energy becomes more negative after their introduction. On the other hand, the addition of a K or La dopant at the top-surface position increases the surface energy. It is found that the addition of K at sub-surface also increases the surface energy by more than 1.0 Jm^−2^, relative to the undoped surface. However, the K-species causes the most significant increase in surface energy when introduced in the top-surface position. It is also observed from Figure 4 that although the addition of Zr to the surface also causes an increase in the surface energy by ~1.0 Jm^−2^, the location of the Zr species on the surface has no effect. From Figure 4a, it is observed that the effect of surface dopants either raises or lowers the surface energy. This suggests the design possibility of using surface additives as dopants to make the nickel catalyst surface either more or less thermodynamically stable relative to the pristine surface. Nonetheless, all the dopants that cause a fluctuation within Δ = ± 0.01 Jm^−2^ in the surface energy relative to the free energy of the pristine surface are considered to have no disruptive thermodynamic effect on the surface.

Figure 4b reveals the role of the dopant on the correlation between the Ni(211) surface energy and the dopant binding energy in the absence of a carbon adsorbate carbon species on the surface. It is immediately clear that the following insights can be gained: Firstly, there is a strong linear correlation between the surface energy and the dopant binding energy—especially for dopants in the sub-surface position. This linear correlation is used as basis for developing a design optimization strategy for Ni catalyst surfaces. This strategy informs the rational determination of appropriate surface dopants to inhibit carbon laydown. Secondly, the effects of Ga, Zr and Sc dopants do not show any sensitivity to their geometrical position on the surface. Thirdly, Zr and Sc dopants caused an increase in surface energy, making it less stable thermodynamically, while Ga decreases the surface energy. Lastly, majority of the dopants only cause a minimal variation in the surface energy when evaluated relative to the undoped surface. It is, therefore, necessary to obtain a holistic picture of the effect of the dopants on the catalyst step surface. This is achieved by investigating the effect of dopants on the adsorption energy of the C_1_ species on the Ni(211) step surface, as well as by exploring the correlations between the dopant-modified free energy of the Ni(211) step surface and the C_1_ adsorption energy. In the following discussion, some crucial trends are shown to emerge from the analyses of the carbon adsorption model on nickel. These emergent trends yield insights for understanding catalyst coking.

**Figure 5:**
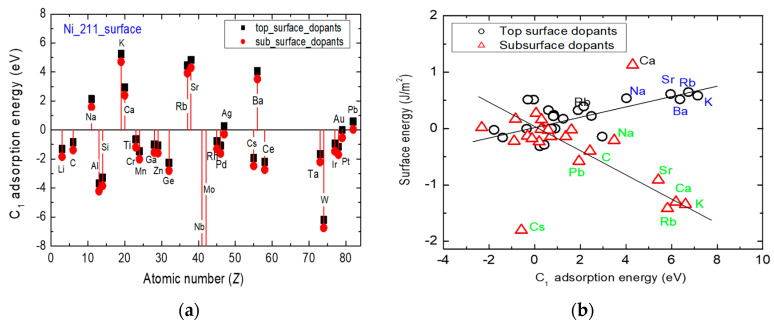
Disruptive effects of single-atom dopants on the free energy of the Ni(211) surface for top-surface (black squares) and sub-surface (red spheres) doping relative to the undoped surface. This figure shows the variation in the C_1_ adsorption energy with the atomic number of the dopant (**a**) and the correlation between the dopant-induced change in the Ni(211) surface energy and the C_1_ adsorption energy (**b**).

Figure 5 shows the correlation between the thermodynamics of carbon nucleation and the disruptive effects of dopants on the adatom adsorption energy on the Ni(211) surface. The preferential adsorption of the C_1_ species occurs at the SH site of the top-surface. An equivalent high symmetry site below the first Ni atomic layer of the step surface was also investigated. This configuration is referred to as the sub-surface doping. Firstly, a negative (positive) adsorption energy of the C_1_ species means that nucleation is thermodynamically favorable (unfavorable) relative to the undoped surface. A comparison of Figure 4a and Figure 5a reveals that dopants have a similar disruptive effect on the free energy of the Ni(111) and Ni(211) surfaces. Figure 5a shows that the introduction of subsurface Nb or Mo reduces the adsorption energy of C_1_ species on the Ni(211) surface by more than 8 eV. The addition of either Nb or Mo to the adsorbate-free Ni(111) decreases the surface energy by more than 4.0 Jm^−2^ [see Figure 4a]. The addition of W is observed to also decrease the C_1_ adsorption energy by 6 eV. It can, therefore, be concluded that none of W, Nb, or Mo are effective inhibitors of the laydown of the C_1_ species on nickel.

Secondly, the addition of K, Rb, Sr, and Ba dopant species to the surface has the opposite effect of increasing the C_1_ adsorption energy by at least 4 eV. Top- and sub-surface doping with Rb, K, and Sr lead to positive increases in the surface free energy and the C_1_ adsorption energy, respectively. In this case, a positive-valued adsorption energy means it is not energetically favorable for the first C_1_ species to nucleate when compared to the adsorption on the SH site of the undoped Ni(211) step surface. Other species, such as Ag, Au, or Pb, have an ignorable effect as dopants. The effect of addition of species K, Rb, Sr, and Ba on the surface suggest that they could be used as surface promoters. Deeper insights can be gained into the surface promotion capability of the dopants by further analyses of the trends obtainable from the surface energy dependence on the C_1_ adsorption energy (see Figure 5b). The effect of doping with K is consistent with its role in potash-based promoters. Other possible candidates exist relative to the K-based promoters.

**Figure 6:**
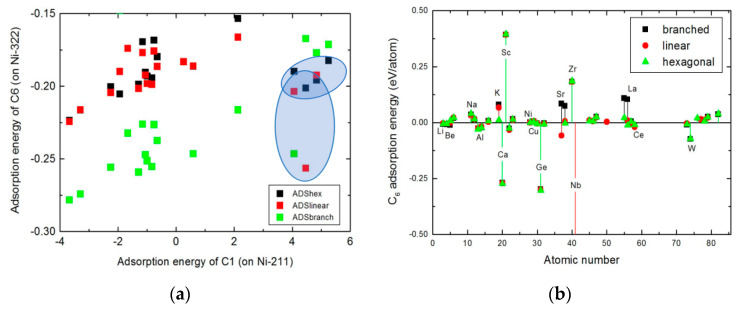
The correlation between the adsorption energies of C_6_ fragments of hexagonal, linear, and branched geometries and the single adatom (C_1_) species (**a**). Influence of surface dopants on the adsorption energy of polymerized C_6_ cluster (**b**).

Two distinct trendlines emerge from the effects of dopants on the carbon adatom adsorption energy on Ni(211) surface if Ca is ignored as the outlier. These disruptive trends correspond to the top- or sub-surface doping types. It is important to emphasize that the zero-energy reference corresponds to the undoped surface. Clearly, the zero-energy reference shown in Figure 6b corresponds to the intersection of the two trendlines. Most of the other dopants have minimal disruptive effect on the surface. Hence, the energy changes when they induce cluster around the zero-energy point. Overall, it does not matter whether the doping is at top-surface or at sub-surface. The same group of dopant species are identifiable with blue and green colors as the ideal modifiers to nickel catalyst surfaces.

#### 3.2.2. Relationship with Other Models of Carbon Formation on Metals

The above mechanistic model of the early stages of the coking process is consistent with the observations of other related studies of the carbon formation on nickel. For instance, Saadi et al. [31] found that graphene grows preferentially out from surface step edges onto lower facets on fcc and hcp metal surfaces. They also found that the epitaxial lattice match between graphene and the metal step-edge stabilizes the graphene and lowers the corresponding critical size of the graphene nucleus. These important insights notwithstanding, the mechanism of carbon nucleation during the initial stages of the growth of epitaxial graphene shows contrasting behavior on stepped metal surfaces [32]. Firstly, the step edges on Ir and Ru surfaces cannot serve as effective trapping centers for single carbon adatoms because two nearby C adatoms repel each other on flat surfaces of Ir(111) and Ru(0001) but preferably form the C-C dimer on Cu(111). 

These contrasting behaviors have been attributed to the delicate competition between C-C bonding and C-metal bonding. The present study has provided deeper insights into these atomistic effects by studying the effect of carbon branching and hexagonal ring formation on the thermodynamic stability of polymeric carbon on nickel surface. Secondly, since carbon nucleates preferentially on Ni surfaces, the present computational studies unravel the origin of the apparent controversy in the laydown mechanism for isolated carbon adatoms on the Ni surface when compared to Ir and Ru surfaces [33]. The controversy arises because although the SH sites of the Ni step edges favor the nucleation of single carbon adatoms, it is ineffective in trapping single carbon adatoms on Ir and Ru surfaces, which readily forms carbon dimers by contrast. 

Li et al. [34] studied up to six carbon atom intermediates (C_6_) on the Ni(111) surface to understand the nucleation of carbon clusters on transition metal substrates and its polymerization during chemical vapor deposition synthesis. They found that carbon chains have higher mobility than branched configurations. The interaction energy between carbon clusters and the Ni surface shows that branched carbon clusters have stronger interaction with the Ni substrate when compared with the carbon chains. Longinova et al. [35] used experimental characterization methods to find evidence for the growth of the graphene sheet by carbon cluster attachment. Their results showed that C adatoms experience a large energy barrier to being attached to the graphene step edges. These led to the conclusion that surface diffusion of the C adatom does not limit the growth of the graphene on the surface.

Table 1 shows a comparison of the carbon cluster sizes and adsorption energy in the mechanistic models considered herein for the laydown of carbon on different facets of the nickel surface relative to previous studies. The asterisk (*) on the adsorption energies in Table 1 denotes energies obtained relative to a single carbon atom (C_1_) that is incorporated into an isolated infinite graphene layer [31]. The corresponding surface coverage (in ML) denotes the ratio *N/N_STEP_* where *N* denotes the number of adsorbed *C_n_* atoms per unit cell and N_STEP_ is the total number of step-edge atoms per unit cell. By contrast, the adsorption energies calculated in this study are obtained from C_n_ species adsorption relative to the clean nickel surface, as specified in Equation (2). These demonstrate the importance of the present calculations and its performance in developing underpinning insights into the coking of the nickel catalyst compared to previous studies.

Previous attempts at using first principles calculations to understand the nucleation, growth, and polymerization of carbon in methane steam reforming reactions was focused on the investigations of the laydown and gasification of monomeric carbon [36]. The present study considers the subsequent polymerization reaction during the carbon laydown. The above mechanistic model is valid for the low-coverage laydown of carbon. The results show that the formation of repeated six-membered rings is preferred to branching. Thus, whenever Ni step sites are still available, carbon will always nucleate from monomeric C_1_ species that are isolated on the nickel surface from methane decomposition. Since carbon formation cannot be avoided when Ni is the metal nanoparticle catalyst of choice, this raises the following question: is there a critical size of the carbon cluster in methane steam reforming? Since methane steam reforming requires a single C_1_ species on the surface to proceed, it is important to understand how the criticality of the cluster size should be defined without violating the geometrical constraints of the surface structure. 

It is shown in the following analyses, that the critical cluster size is made of four carbon atoms for nickel catalyst coking to be spontaneous. In Section 3.3, it is shown that there is no critical size of the carbon cluster for the facile burn-off of polymeric carbon in MSR. To gain insights into possible answers to this question, adsorption energies of larger carbon fragments have been calculated on the larger Ni(322) surface for the carbon clusters of increased size. The growth of C_6_ species on nickel is modeled here as hexamer adsorption on Ni(322). The calculated hexamer adsorption energies on Ni(322) step surface are used to clarify carbon nucleation on growth on typical catalysts—from adatoms to graphene.

Figure 6 shows the interrelationship (if not a correlation) between the adsorption energy of C_6_ cluster of hexagonal, linear, and branched shape and the adsorption energy of a single adatom (C_1_) species and the influence of surface dopants on the adsorption energy of polymerized C_6_ cluster. A linear trendline is seen to emerge independent of the local geometry of the carbon clusters if the outlying data points that are indicated by the blue-shaded elliptical rings are disregarded in Figure 6a. This reveals that a strong correlation exists between the adsorption energies of the C_1_ and C_6_ fragments. Figure 6b shows that Nb doping favors the formation of linearly polymerized carbon chain, while Sc and Zr inhibits the formation of hexagonal rings. Crucially, the K species still shows effectiveness as an inhibitor of the hexamer species adsorption; its effect is not as dominant as the case in Figure 4 and Figure 5.

**Figure 7:**
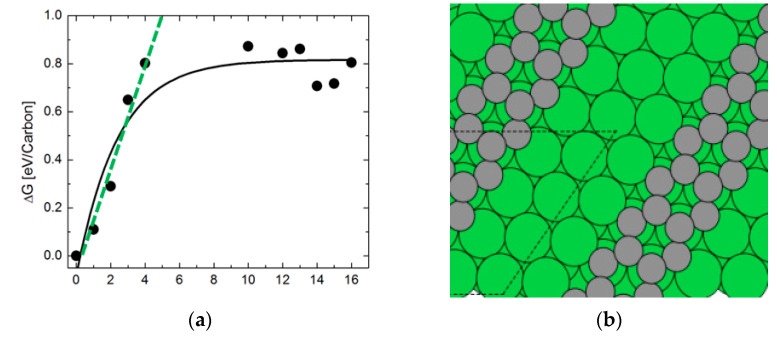
Thermodynamic reaction profile of the polymeric carbon laydown on Ni(322) surface in methane steam reforming (**a**) and the supercell model of the local surface structure of a contiguous layer of carbon showing a partially-coked Ni(322) surface model of the catalyst (**b**).

Figure 7 shows the changes in the Gibbs free energy (ΔG) of the deposited polymeric carbon as a function of C_n_ up to *n* = 16 and the supercell model of local surface structure of a contiguous carbon fragment containing 16 carbon atoms in hexagonal geometry. This thermodynamic model is useful for understanding the coking of the nickel step surface of the catalyst surface. Firstly, it observed that the profile of the Gibbs free energy for the polymerization reaction has two distinguishable trendlines. The first region shows a rapidly increasing ΔG as the size C_n_ of the carbon cluster increases from 1 up to 5. By fitting a linear trendline to the fluctuations in the ΔG as C_n_ changes locally within the first region, the critical size of the carbon cluster, which is necessary for the spontaneous coking of the nickel catalyst during methane steam reforming is C_5_. By contrast, the second region shows a nearly constant ΔG wherein the free energy of polymerization is insensitive to carbon cluster size. In this latter region, the local structure of the polymeric chains favor the formation of hexagonal rings. Thus, carbon adsorption energies decrease with increases in carbon cluster size. This suggests that the polymerization process is thermodynamically limited when the carbon cluster contains more than five carbon atoms.

### 3.3. Rational Promotion of Carbon Burn-Off in Methane Steam Reforming

#### 3.3.1. Facile Routes for Monomeric Carbon Burn-Off

Apart from using surface dopants to inhibit carbon laydown, the lattice structure of the Ni alloy can be expanded to induce strain in the carbon overlayer. These two strategies rely on the modification of the electronic structure to hinder the growth of the carbon overlayer. It is also possible to rationally promote the burn-off of carbon. Since the stripping reaction for the dehydrogenation of methane must be completed first before the first C_1_ species can be adsorbed at the active surface site on the catalyst, then a fully efficient monomeric burn-off of the C_1_ species is the only way to guarantee that carbon does not buildup on the surface. This level of efficiency is counterintuitive experimentally since nickel catalyzes coke. Thus, the potential energy surface at 0 K is used to gain insights to the possible routes for carbon gasification. 

**Figure 8:**
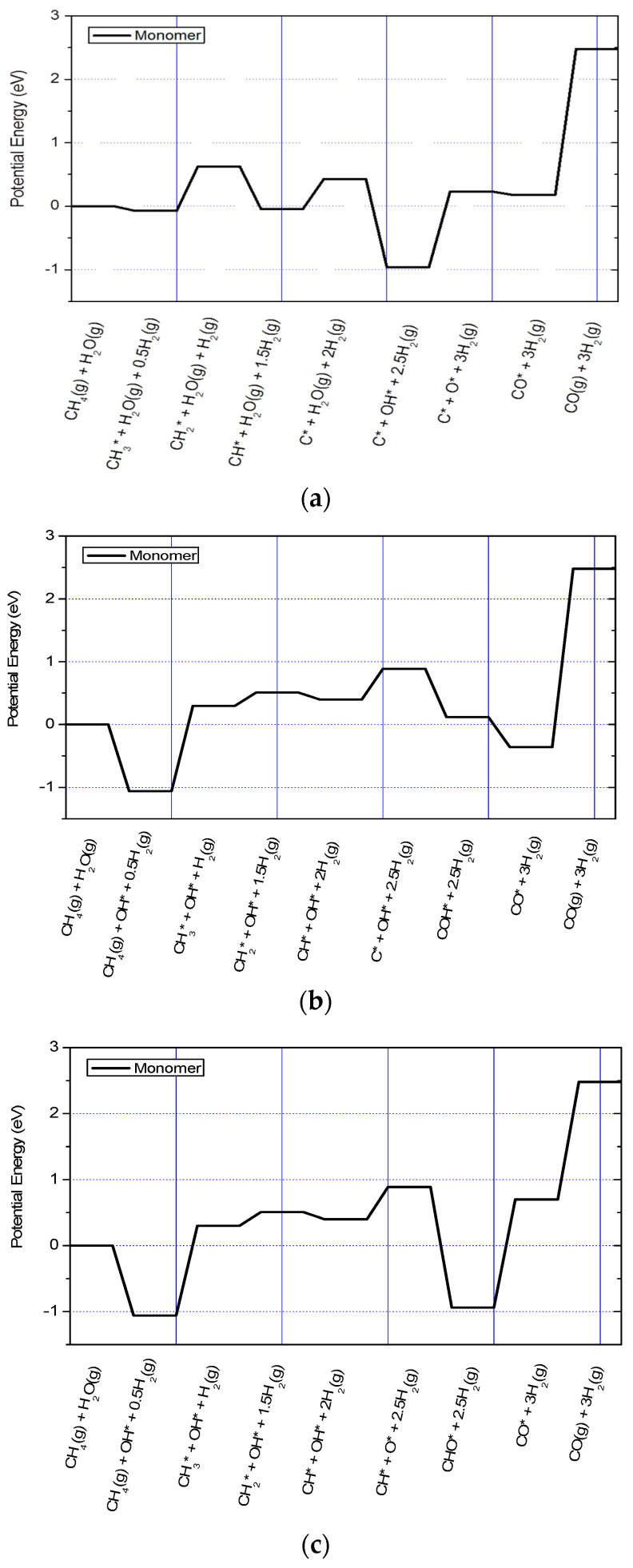
The 0 K potential energy profile of the CO*(**a**), COH*(**b**), and CHO*(**c**) routes for the burn-off of monomeric carbon modelled on the Ni(322) step surface.

Figure 8 shows the full potential energy profile of the three routes for the monomeric carbon laydown and burn-off mechanism modelled on Ni(322) step surface. The 0 K potential energy profiles are shown at different intermediate steps of the laydown and burn-off process. This gives insights into the role of the electronic structure at zero temperature. This is an important distinction since the methane steam reforming requires a high temperature to drive. The carbon adatom must be present on the nickel surface to form O* and H_2_ species. There are important differences between the mechanisms of the three routes for in situ monomer burn-off. 

**Figure 9:**
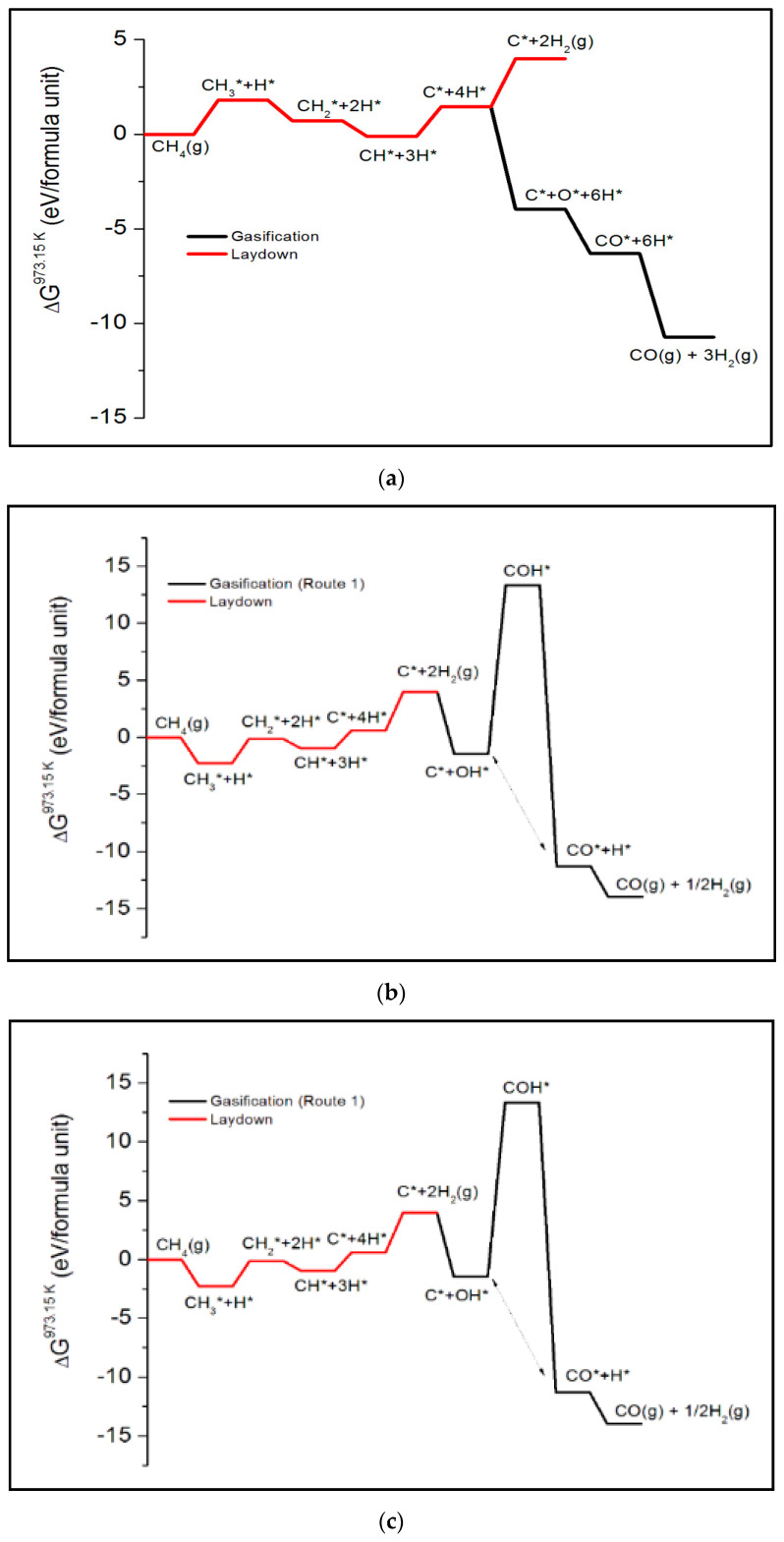
Thermodynamic reaction profile at 973 K showing the CO*(**a**), COH*(**b**), and CHO*(**c**) routes for the monomeric carbon burn-off mechanism modelled on the Ni(322) step surface.

Firstly, in the CO* route, surface carbon species C* forms. The surface OH* only forms from the steam after the laydown of the monomeric C* species on the surface. It is the surface C* species that reacts with the OH* species and molecular hydrogen to yield the carbon monoxide in the gas phase, according to Figure 9a. Secondly, in the COH* route (see Figure 9b), the surface OH* species form from steams first before the methane dehydrogenation reaction for the monomeric carbon laydown completes CH_4_ (*g*) → C* + 4H*. Thirdly, the methane decomposition reaction is incomplete in the CHO* route (see Figure 9c). Hence, there is no laydown of the monomeric C* species. This is because methane dehydrogenation stops at the formation of the surface CH*. It is the CH* that reacts with the surface O* and OH* species, leading to the formation of CHO* on the surface. The combined methane steam reforming reaction of each route has a change in the total energy of ~2.5 eV, indicating the endothermic nature of the process.

Figure 9 shows the effect of including entropic contributions to the thermodynamic free energy at 973.15 K. Although the effect of steam has not been included in the analysis of the methane cracking reaction for the monomeric carbon laydown, it is observed that the free energy profiles in Figure 9a–c show that the reaction is exothermic and proceeds spontaneously at high temperature. Although the intermediate step for the COH* formation (see Figure 9b) has a high thermodynamic barrier, all the subsequent intermediate steps are energetically favorable. In this case, the increased temperature of the simulated reaction makes the methane steam reforming reaction become overwhelmingly favorable thermodynamically.

#### 3.3.2. Role of Steam in the Burn-Off of Polymeric Carbon

Carbon polymerization was modelled in Section 3.1.2 as the addition of monomers to an existing polymeric chain without including the effects of steam. However, the methane steam reforming reaction for the formation of surface [C_n_]* species from [C_1_]* + [C_n−1_]* requires the dissociation of water, in addition to the methane cracking. The presence of water at high temperature favors the laydown of [C_n_]* species because of the negative Gibbs free energy of the reaction. For instance, ΔG << 0 (see Figure 9). The high-temperature dissociation of water to form either O* or OH* species only takes place in the presence of the polymeric [C_n_]* species on the nickel surface. This intermediate step is crucial in inducing the gasification through carbon oxidation C* + O* → CO* or C* + OH* to release H_2_ species in each case. Thus, the polymeric carbon laydown is understandable as the two-step reaction model that combines methane cracking (Step 1) with the dissociation of water (Step 2) as coupled reactions:

*n*CH_4_ → [C_n−1_]* + [C]* + 2*n*H_2_ + [C_n_]*, (8)

*n*H_2_O + [C_n_]*→ [O]* + H_2_ + [C_n_]*. (9)

**Figure 10:**
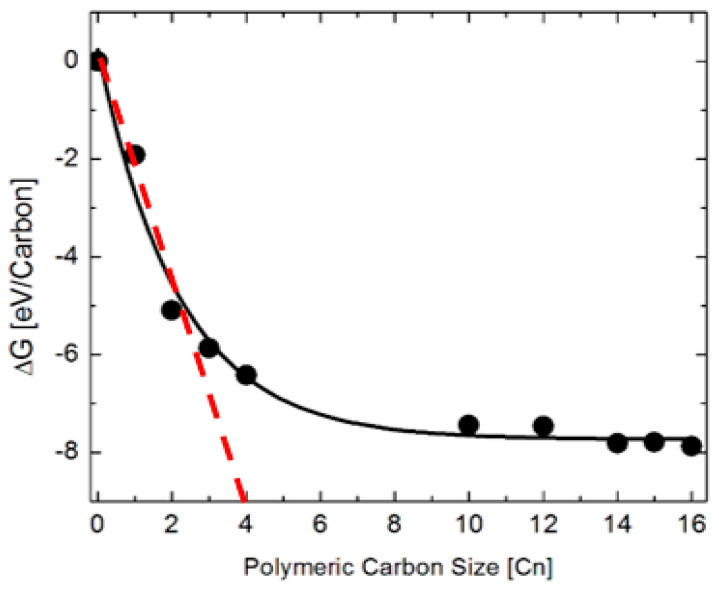
Variations in the change in Gibbs free energy with size of the polymeric carbon cluster in the water dissociation reaction at 973.15 K.

Figure 10 shows the change in free energy of the water dissociation reaction at 973.15 K for a variable size of the polymeric carbon. The exponential decay profile of the Gibbs free energy change during the carbon polymerization reaction has two distinguishable trendlines. In this case, the first region shows a rapidly decreasing ΔG as the cluster size C_n_ increases from 1 up to 4. A linear fit trendline is provided for the fluctuations in the ΔG as C_n_ changes within the first region, and the critical size of the carbon cluster increases, which is necessary for the spontaneous dissociation of steam during methane steam reforming in C_4_. In the second region, by contrast, the change in free energy is insensitive to the size of the carbon cluster. Figure 10 shows a nearly constant ΔG of ~−8 eV/carbon in the limit of large carbon clusters, where the size is C_n_ → C_16_.

#### 3.3.3. Facile Routes for Polymeric Carbon Burn-Off

**Figure 11:**
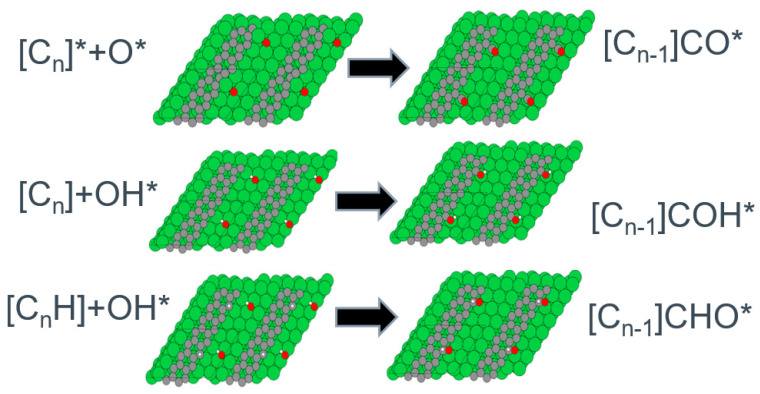
Ball and stick model of the reactants and products in the reactive burn-off of polymeric carbon by direct carbon monoxide, hydroxyl, and formyl formation routes.

Figure 11 shows the ball and stick model of the reactants and products in the three routes for implementing routes for polymeric carbon burn-off in methane steam reforming. These include the promotion of the direct formation of surface CO*, hydroxyl (CHO*), and formyl (CHO*) species.

**Figure 12:**
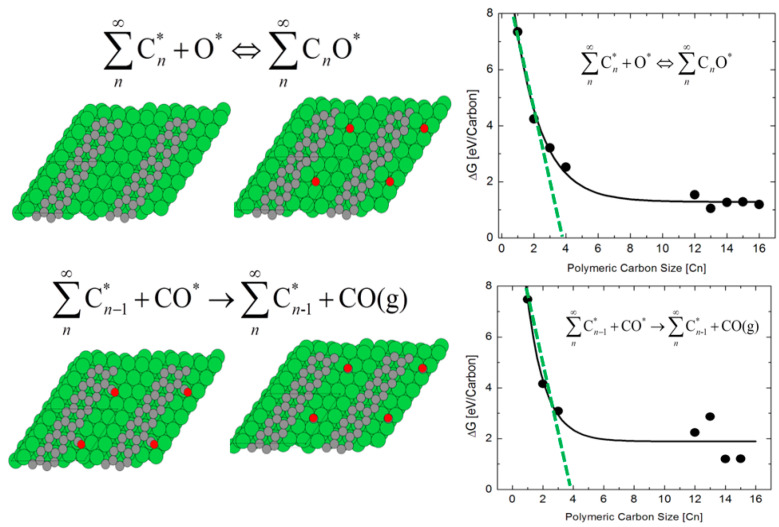
Intermediate steps in the direct carbon monoxide formation mechanism and their free energy profiles for implementing a reactive burn-off.

Figure 12 shows the intermediate steps and the corresponding free energy profiles for the direct carbon monoxide formation mechanism for implementing a reactive burn-off. The burn-off reaction pathway occurs via C_n_O* formation, according to the reaction:

[C_n_]* + [O]* ↔ [C_n_O]* (10)

It is observed that a similar correlation exists between the free energy of reaction for the burn-off of polymeric carbon and the size of the carbon cluster, as observed for the water dissociation reaction (see Figure 10). Two energy regimes are identifiable in the formation of C_n_O* and C_n−1_* + CO(g) intermediates. In both reactions, the regions wherein C_n_ ≤ 4 and C_n_ > 4 exhibit different thermodynamic profiles are energetic. A linear fit to the dependence of ΔG on Cn yield a linear dependence with *n* = 4 as the critical size of the cluster. Thus, it is seen that the free energies required to drive the burn-off are correlated with carbon polymerization up to the critical cluster size of the carbon tetramer (C_4_). These correlations suggest that dopants will have the same disruptive effects on polymeric carbon laydown as in the monomeric case. As surface dopants, it is seen that introduction of Nb, Mo, Ga, Ca, Ag, and Ta will aid catalyst coking, while the introduction of Sc, Sr, Zr, Ba, K, Cs, and Zn will likely promote the facile burn-off of any coke deposits.

## 4. Conclusions

In summary, first principles calculations have been performed based on density functional theory to study the mechanistic models of the laydown of monomeric and polymeric carbon species on nickel surface facets, and models of their burn-off mechanism. This has allowed for the rational identification of ways to suppress the laydown of carbon species on the nickel surface while simultaneously allowing the burn-off of surface carbon species to occur before they can polymerize. This study allows for insights into the reaction pathways for catalyst coking to be gained. Materials development strategies that favor the modification of the catalyst surface with single atoms species are proposed to inhibit carbon laydown and promote carbon burn-off. This strategy is based on the targeted surface modification with a dopant species that serve either as a selective inhibitor of the surface adsorption of the polymerizing carbon or facilitator of the burn-off. Insights are gained into the reaction mechanisms that culminate in the coking of a nickel catalyst. Routes for the gasification of the polymerized carbon species are explored for the recovery of a nickel catalyst during the steam reforming of methane for the large-scale production of hydrogen. The insights developed herein are expected to fill currently existing gaps in experimental and computational data on the coking models of the nickel catalyst, and to guide experiments towards a more effective design of nickel-based catalysts.

## Figures and Tables

**Table 1:** Carbon cluster sizes and the corresponding adsorption energy in the laydown of carbon on different facets of the nickel surface. An asterisk (*) denotes adsorption energies that are obtained relative to a single carbon atom (C_1_) that is inserted into an isolated infinite graphene layer [31].

Size, C_n_	Ni Surface	Adsorption Energy (eV)	Laydown Model
C_1_ (at 0.5 ML coverage)	Ni(100)	0.25 * [31]	Monomeric
C_1_ (at 1.0 ML coverage)	Ni(100)	0.70 * [31]	Monomer
C_1_	Ni(111)	1.25 * [31]	Monomeric
C_1_	Ni(211)	−0.95 [This work]	Monomeric
C_2_	Ni(111)	−0.56 [34], −0.58 [This work]	Polymeric
C_2_	Ni(211)	−0.89 [This work]	Monomeric
C_3_	Ni(111)	−0.85 [34]	Polymeric
C_3_	Ni(322)	−0.88 [This work]	Monomeric
C_4_	Ni(111)	−1.59 [34], −1.71 [This work]	Polymeric
C_4_	Ni(322)	−1.88 [This work]	Polymeric
C_4_–C_6_	Ni(322)	−1.94 [This work]	Polymeric
C_6_–C_10_	Ni(322)	−2.02 [This work]	Polymeric
C_10_–C_16_	Ni(111)	−2.08 [This work]	Polymeric

## Data Availability

The data presented in this study are available within the article.
